# Most plant-based meat alternative buyers also buy meat: an analysis of household demographics, habit formation, and buying behavior among meat alternative buyers

**DOI:** 10.1038/s41598-022-16996-5

**Published:** 2022-07-29

**Authors:** Zachary T. Neuhofer, Jayson L. Lusk

**Affiliations:** Department of Agricultural Economics, Purdue University, West Lafayette, IN USA

**Keywords:** Environmental economics, Psychology and behaviour, Human behaviour

## Abstract

The promise of novel plant-based meat alternatives (PBMAs) to lessen the health and environmental impacts of meat consumption ultimately depend on market acceptance and the extent to which they displace meat in consumers’ diets. We use household scanner data to provide an in-depth analysis of consumers’ PBMA buying behaviors. PBMAs buyers tend to be young, single, female, college educated, employed, higher income, and non-white. About 20% of consumers purchased a PBMA at least once, and 12% purchased a PBMA on multiple occasions. About 2.79% of households only purchased PBMAs. About 86% of PBMA buyers also bought ground meat; however, PBMA buyers spent about 13% less on ground meat. Interestingly, after a household’s first PBMA purchase, ground meat consumption did not fall. The number of households buying a PBMA for the first time fell over the two year period studied, despite the increase in market share in the ground meat market.

## Introduction

In recent years, concerns have increased around the health and environmental impacts of meat consumption^1–8^. Encouraging dietary change toward more plant-based diets has been proposed to reduce dietary-related land-use, water use, and greenhouse gas emissions^1,2,9,10^. From a public health perspective, substitution to a plant based diet has been estimated to lower the risk of chronic diseases and mortality^11–14^. Despite encouragement for dietary change, consumer demand for meat remains high and is growing^15^. Moreover, there is little public support for policies to tax foods based on health or environmental outcomes^16^. The current dilemma faced by the industry and policymakers is between persistently high meat demand and a lack of political support for policies that would curb meat consumption. A potential solution is to invest in innovative plant-based meat alternatives (PBMA) that have the potential to induce consumers to willingly transition away from animal to plant-based diets^17–21^. Novel PBMAs use ingredients such as soy, wheat, pea, and heme to mimic the sensory characteristics and macronutrients of meat.

Interest in PMBAs also stems from potential environmental benefits. Several studies have suggested that reducing meat consumption in favor of a more plant-based diet will lead to better environmental outcomes, which include, reducing greenhouse gas (GHG) emissions, land use, and water use relative to conventional beef production^3,17,18,22,23^. However, for these environmental benefits to be realized, consumers must be willing to choose PBMAs over meat options. At this point, much remains unknown about consumer purchase behaviors surrounding PBMAs.

What is known about consumer preferences for PBMAs is primarily limited to hypothetical stated preference surveys^16,24–27^, industry and interest groups reports^28,29^, and analysis of retail scanner data that aggregates over thousands of consumers^30^. Analysis of household scanner data has only recently been undertaken^31^. These previous studies have estimated market shares for PBMAs that range from 30% to less than 1%. These estimates depended on how the market is defined (e.g., ground products vs. all beef vs. all meat), the data source used (e.g., stated preference survey vs. retail scanner data), and the unit of measurement (e.g., share of quantities sold or share of sales)^16,26–30,32,33^. Most estimates of retail scanner data put the dollar market share of PBMA out of all meat sales at less than 1%, a figure that grew rapidly from 2019–2021, but that appears to have stalled in 2022^28,30^. Analysis using scanner data has suggested low repeat purchases of PBMAs and that PBMAs are not a substitute for beef or pork^31^.

Some studies have analyzed the demographic characteristics of PBMA buyers^16,24–26,31,32,34,35^. These studies found that PBMA consumers are more likely to be younger, higher income, female, more educated, politically liberal, residing in urban areas, and with young children^16,24–26,31,32,34,35^. These prior studies also identified key consumer beliefs associated with PBMA consumption, such as, following a vegetarian or vegan diet, concerns for the environmental impact of meat production, health concerns of meat consumption, and more concern for animal welfare^16,24–26,36–38^.

The primary objective of this study is to determine dynamics of the PBMA market and consumer characteristics of PBMA consumers using household scanner data spanning the two year period of November 2018 to November 2020. These rich transaction-level data help inform a number of key unresolved questions about the demand for PBMAs. What are the drivers of PBMA purchases? We expect that demand for PBMAs is stable and growing if consumers are particularly concerned for the environmental impacts of meat production and animal welfare, while being satisfied with the taste of PBMAs. If instead, however, novelty is a key driver, high initial sales may be followed by stagnation in sales, once the novelty of the PBMA products has worn off. Insights into this matter can be explored by determining the extent which PBMA sales are coming from repeat vs. new household purchases. Another key question is the extent to which consumers are substituting away from meat toward PBMAs, in which case the new products are likely to hamper meat demand. Also, if sales of PBMAs are coming from households who do not primarily purchase much meat, the rising market share of PBMAs could be explained by an expansion of the market. Finally, what are the characteristics of people who buy PBMAs? Knowledge of such information can help identify target markets and provide insights into the trajectory of sales.

## Results

### Market share

**Figure 1:**
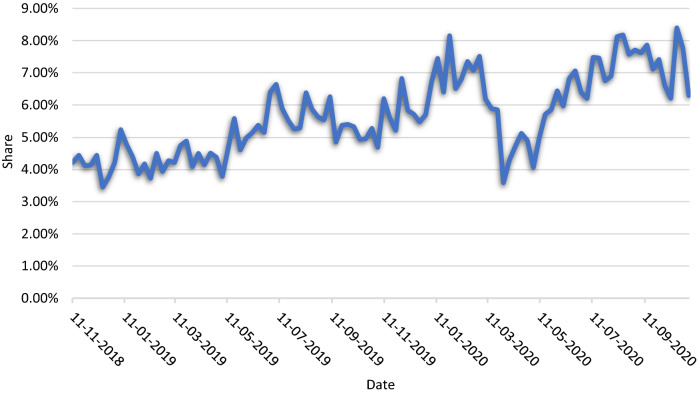
Plant-based meat alternative share of total expenditures on ground meat products.

Out of sales for all ground meat products, the PBMA expenditure share averaged 5.91% from November 2018 to November 2020 (Fig. 1). The PBMA expenditure share gradually increased over the two year span, reaching a peak of 8.40% in October of 2020, nearly double the initial 4.22% share in November of 2018. The average PBMA expenditure experienced a sharp but brief decline after the onset of the COVID-19 pandemic in March and April of 2020 but then recovered in May of 2020 (Supplementary Fig. 1).

### General household behavior

**Figure 2:**
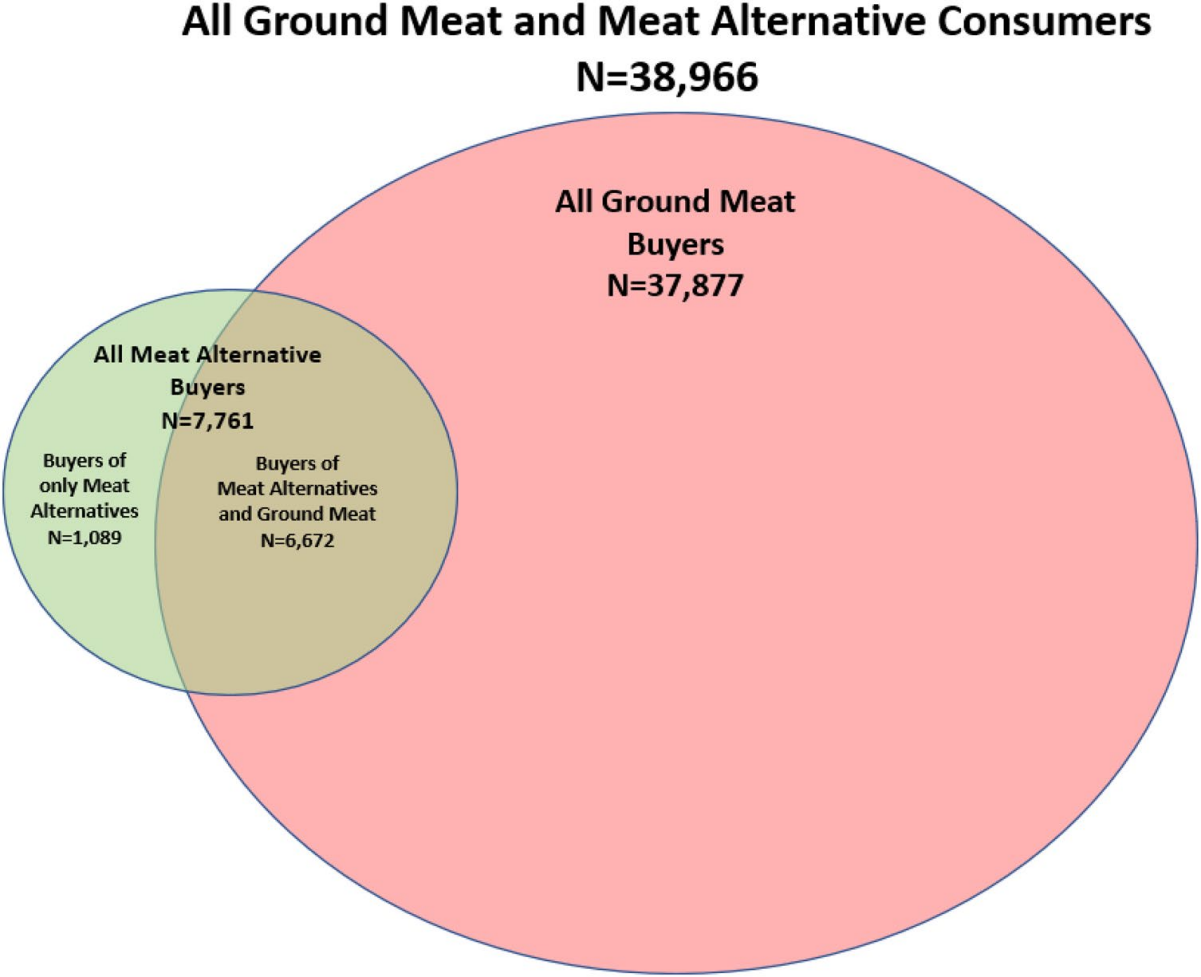
Overview of household consumption behavior.

Over the two year period 7,761 (19.92%) households purchased a PBMA at least once, while 31,205 (80.08%) households did not purchase a PBMA over this time (Fig. 2). Among the full sample of consumers, 12.03% of households bought PBMAs on multiple occasions (Supplementary Table 2). Among households who purchased a PBMA at least once, 60.15% made a repeat purchase, meaning 39.85% of PBMA buyers tried the product only once.

In the full sample of households, a small fraction 1,089 (2.79%) avoided ground meat entirely and only purchased PBMAs (Fig. 2). Of consumers who bought a PBMA, 14.51% did not purchase ground meat. Most households (85.97%) that purchased a PBMA also purchased ground meat at some point in the two year span; however, as will soon be noted, PBMA buyers tended to spend less on ground meat than non-PBMA buyers.

### Demographic analysis of household behavior and market analysis

Table 1 shows that a larger share of households with a younger head (< 35) were more likely to make a PBMA purchase than middle age (35–64) and older households (65 +). A significantly higher share of single female households purchased a PBMA than married or single male households. Additionally, a higher share of households that have an employed or college educated household head purchased a PBMA. Significant differences were also observed among income groups, revealing households with an income of $100,000 or greater were more likely to purchase a PBMA than middle income ($45,000-$99,999) or lower income (< $45,000) households. A larger share of African-American and other minority households purchased a PBMA than white households. Also, larger share of households with children purchased a PBMA than households without children.

**Table 1:** Meat alternative buying behavior by demographic category.

Variable	Category	Share of meat alternative buyers (Full Sample) (%)	Share of meat alternative buyers who purchased them on more than one occasion (Full Sample) (%)	Share of meat alternative buyers who purchased them on more than one occasion (Meat Alternative Buyers) (%)	Share of households that did not purchase meat alternatives (Full Sample) (%)	Share of households who purchased meat and meat alternatives (Full Sample) (%)	Share of households who purchased meat and meat alternatives (Meat Alternative Buyers) (%)	Share of households that did not purchase meat (Full Sample) (%)	Share of households that did not purchase meat (Meat Alternative Buyers) (%)
Age	< 35	25.13**	15.19**	60.43*	74.87**	21.15**	84.14	3.99**	15.86
	35–64	21.35**	13.09**	61.30*	78.65**	18.48**	86.56	2.87**	13.44
	65 +	16.33**	9.49**	58.11*	83.67**	13.88**	85.02	2.45**	14.98
Household Size	1	21.14**	12.76**	60.39	78.87**	15.94**	75.43**	5.19**	25.57**
	2	18.63**	11.29**	60.61	81.37**	16.41**	88.07**	2.22**	11.93**
	3	21.17**	12.91**	60.99	78.83**	19.16**	90.47**	2.02**	9.53**
	4	21.69**	13.13**	60.55	78.31**	19.67**	90.71**	2.01**	9.29**
	5 +	19.13**	10.96**	57.30	80.87**	17.66**	92.32**	1.47**	7.68**
Marital Status	Married	19.34**	11.72*	60.60	80.66**	17.38**	89.86**	1.96**	10.14**
	Single Female	21.46**	12.83*	59.77	78.54**	17.25**	80.38**	4.21**	19.62**
	Single Male	19.56**	11.90*	60.83	80.44**	14.05**	71.85**	5.51**	28.15**
College Education	No	16.89**	9.82**	58.13**	83.11**	14.67**	86.84*	2.22**	13.16*
	Yes	24.15**	15.11**	62.58**	75.85**	20.55**	85.11*	3.59**	14.89*
Employed	No	16.44**	9.53**	58.00*	83.56**	14.02**	85.31	2.42**	14.69
	Yes	21.31**	13.02**	61.11*	78.69**	18.36**	86.17	2.95**	13.83
Income	< $45,000	16.80**	10.07**	59.94	83.20**	13.79**	82.11**	3.01	17.89**
	$45,000–$99,999	19.78**	11.86**	59.99	80.22**	17.10**	86.47**	2.67	13.53**
	$100,000 +	24.49**	15.04**	61.42	75.51**	21.74**	88.79**	2.75	11.21**
Race	White	18.96**	11.49**	60.59	81.04**	16.36**	86.30**	2.60**	13.70**
	African American	23.47**	14.19**	60.48	76.53**	20.77**	88.52**	2.69**	11.48**
	Other Minority	23.71**	13.95**	58.84	76.29**	19.11**	80.58**	4.60**	19.42**
Hispanic	No	19.81	12.00	60.59	80.19	16.99**	85.79	2.81	14.21
	Yes	21.15	12.31	58.21	78.85	18.56**	87.75	2.59	12.25
Presence of children	No	19.63**	11.85*	60.38	80.37**	16.65**	84.80**	2.98**	15.20**
	Yes	21.08**	12.73*	60.39	78.92**	19.04**	90.31**	2.04**	9.69**

Chi-Square tests were used to test the differences in group probability between each demographic category with the null hypothesis stating there is no association between the demographic category and the outcome. The sample used for the statistical test is denoted in parenthesis in the column description. A * denotes significance at the 5% level and **denotes significance at the 1% level.

A majority of consumers did not purchase PBMAs at all. Non-PBMA buyers were more likely to be older (65 +), married or single males, not college educated, not employed, lower income (< 45,000), white, and without children, rather than younger (< 35) or middle age (35–64), single females, college educated, employed, middle ($45,000–$99,999) or high income (> $100,000), non-white racially, and without children.

About 12% of households (6,670), purchased both ground meat and PBMAs over the two year period. A higher share of dual-purchasing households is younger (< 35), married or single females, college educated, employed, high income (> $100,000), African American, Hispanic, and with children present, relative to dual-purchasing households that are middle age (35–64) and older (65 +), single males, non-college educated, unemployed, low (< $45,000) or middle income ($45,000–$99,999), white or other race, non-Hispanic, and without children present. Among the sample of PBMA buyers, a majority purchased both PBMA and ground meat, but a higher share of households with more members (≥ 2), married, not college educated, high income (> $100,000), African-American, and with children present, purchased both PBMAs and ground meat relative households that were smaller (< 2), unmarried, college educated, lower (< $45,000) or middle income ($45,000–$99,999), white or other race, and without children present.

Some households (1087) did not purchase any ground meat (Fig. 2). This group has a higher share of households that are younger (< 35), with one member, single males, college educated, employed, a minority that is not African-American, and without children, relative to households that are middle age (35–64) and older (65 +), with more than one member, single females or married, non-college educated, unemployed, white or African American, and with children present. In the sample limited to PBMA buyers, a higher share of households with one member, single male heads, college educated, low income (< $45,000), of a minority that is not African American, and without children present only purchased PBMAs relative to households with more than one member, single females or married, not college educated, middle ($45,000–$99,999) or high income (> $100,000), white or African-American, and with children present.

A higher share of households that are younger (< 35), single females, college educated, employed, higher income (> $100,00), African-American and other minorities, and with children present made repeat purchases of PBMAs, relative to households that are middle age (35–64) and older (65 +), single males or married, non-college educated, unemployed, low (< $45,000) or middle income ($45,000–$99,999), white, and without children present. Among the sample of PBMA purchasing households, there were few significant differences among demographic groups in repeat-purchase behavior.

The average household in the full sample purchased 1.53 PBMA units (or 0.01 per week) over the two year span (Table 2; Supplementary Fig. 2). Looking only at PBMA purchasing households, 7.69 units on average were purchased in two years (or 0.07 per week). Among all households, two-year expenditures on PBMAs were $6.78 ($0.07/week) and was $34.04 (or $0.33/week) for households who purchased at least one PBMA. By contrast, households spent $112.74 over the two year period on ground meat ($1.08/week). For households who purchased a PBMA, they spent $97.78 on ground meat ($0.94/week).

**Table 2:** Average Expenditures and Units Purchased.

Metric	Sample	Sample size	Average
**Meat alternative units purchased and expenditure**			
Average number of meat alternative units purchased by a single household	Full sample	38,966	1.53
	Meat alternative buyers	7761	7.69
Average units of meat alternatives purchased weekly by a single household	Full sample	38,966	0.01
	substitute buyers	7761	0.07
Average total expenditure on meat alternatives by a single household	Full sample	38,966	$6.78
	Meat alternative buyers	7761	$34.04
Average weekly expenditure on meat alternatives by a single household	Full sample	38,966	$0.07
	Meat alternative buyers	7761	$0.33
**Meat units purchased and expenditure**			
Average number of meat units purchased	Full sample	38,966	15.95**
	Meat alternative buyers	7761	15.43**
Average units of meat purchased weekly by a single household	Full sample	38,966	0.153**
	Meat alternative buyers	7761	0.148**
Average total expenditure on meat by a single household	Full sample	38,966	$112.74**
	Meat alternative buyers	7761	$97.78**
Average weekly expenditure on meat by a single household	Full sample	38,966	$1.08**
	Meat alternative buyers	7761	$0.94**
**Changes in meat consumption behavior after purchase of first meat alternative**			
Average units of meat purchased weekly prior to the first purchase of a meat alternative by a single household	Meat alternative buyers	6390	0.1 $${6}^{++}$$
Average units of meat purchased weekly after the first purchase of a meat alternative by a single household	Meat alternative buyers	6390	$${0.18}^{++}$$
Average weekly expenditure on meat prior to the first purchase of a meat alternative by a single household	Meat alternative buyers	6390	$$\mathrm{\$}{0.70}^{++}$$
Average weekly expenditure on me after the first purchase of a meat alternative by a single household	Meat alternative buyers	6390	$$$1.2{0}^{++}$$

A *denotes an ANOVA test conducted to compare the means between the meat alternative buyers and remaining sample, with the null hypothesis $\mu_{{{\text{Full}}}} = \mu_{{{\text{Buyer}}}}$. A * denotes statistical significance at the 5% level and ** denotes significance at the 1% level. A + denotes a paired T-test to compare the difference in means of two different variables with the null hypothesis $\mu_{{{\text{after}}}} - \mu_{{{\text{before}}}}$. A + denotes significance at the 5% level and a +  + denotes significance at the 1% level.

The time-series nature of the data permits an investigation of purchase behavior before and after the first observed purchase of PBMAs. The average PBMA purchasing household bought more ground beef units (0.02) and spent more on ground beef ($0.50) after their first PBMA purchase then they did prior to their first PBMA purchase. New entrants into the PBMA market declined over the period (Supplementary Fig. 5) despite the fact that even at the period’s end, 80.08% had not purchased a PBMA. The week in which the highest number of households entered was the initial week of the study period in November 11, 2018.

Significant differences between demographic groups were observed with regards to PBMA units purchased and expenditures (Table 3). In the full sample, the households that purchased the most PBMA units and had the highest expenditures were younger (< 35), single males, college educated, employed, high income (> $100,000), and African-American compared to households that are middle age (45–64) or older (65 +), single females or married, not college educated, unemployed, low (< $45,000) or middle income ($45,000-$99,999), and white or another minority race. Among the sample including only PBMA buyers, single males purchased more units and had higher expenditure than females.

**Table 3:** Meat alternative units purchased and expenditure by demographic category.

Variable	Category	Average number of aggregate meat alternative units purchased (Full Sample)	Average number of aggregate meat alternative units purchased (Meat Alternative Buyers)	Average number of meat alternative units purchased weekly (Full Sample)	Average number of meat alternative units purchased weekly (Meat Alternative Buyers)	Average total expenditure on meat alternatives (Full Sample)	Average total expenditure on meat alternatives (Meat Alternative Buyers)	Average weekly expenditure on meat alternatives (Full Sample)	Average weekly expenditure on meat alternatives (Meat Alternative Buyers)
Age	< 35	2.034**	8.094	0.020**	0.078	$9.10**	$36.19	$0.09**	$0.35
	35–64	1.679**	7.865	0.016**	0.076	$7.43**	$34.79	$0.07**	$0.33
	65 +	1.169**	7.161	0.011**	0.069	$5.16**	$31.62	$0.05**	$0.30
Household Size	1	1.566	7.408	0.015	0.071	$6.60	$31.21	$0.06	$0.30
	2	1.466	7.868	0.014	0.076	$6.50	$34.90	$0.06	$0.34
	3	1.586	7.489	0.015	0.072	$7.14	$33.71	$0.07	$0.32
	4	1.708	7.878	0.016	0.076	$7.77	$35.85	$0.07	$0.34
	5 +	1.475	7.712	0.014	0.074	$6.93	$36.24	$0.07	$0.35
Marital Status	Married	1.500*	7.754**	0.014*	0.075**	$6.78	$35.06**	$0.07	$0.34**
	Single Female	1.518*	7.074**	0.015*	0.068**	$6.45	$30.05**	$0.06*	$0.29**
	Single Male	1.912*	9.776**	0.018*	0.094**	$8.11	$41.43**	$0.08	$0.40**
College Education	No	1.321**	7.820	0.013**	0.075	$5.77	$34.16	$0.06**	$0.33
	Yes	1.827**	7.566	0.018**	0.073	$8.19	$33.92	$0.08**	$0.33
Employed	No	1.185**	7.211	0.011**	0.069	$5.17	$31.44	$0.05**	$0.30
	Yes	1.670**	7.840	0.016**	0.075	$7.42	$34.84	$0.07**	$0.33
Income	< $45,000	1.274**	7.585	0.012**	0.073	$5.33	$31.72	$0.05**	$0.31
	$45,000-$99,999	1.569**	7.936	0.015**	0.076	$6.94	$35.08	$0.07**	$0.34
	$100,000 +	1.812**	7.400	0.017**	0.071	$8.46	$34.54	$0.08**	$0.33
Race	White	1.477**	7.787	0.014**	0.075	$6.37	$33.62	$0.06**	$0.32
	African American	1.832**	7.809	0.018**	0.075	$8.43	$35.93	$0.08**	$0.35
	Other Minority	1.636**	6.901	0.016**	0.066	$8.20	$34.61	$0.08**	$0.33
Hispanic	No	1.542	7.785	0.015	0.075	$6.77	$34.20	$0.07	$0.33
	Yes	1.427	6.745	0.014	0.065	$6.86	$32.43	$0.07	$0.31
Presence of children	No	1.523	7.760	0.015	0.075	$6.69	$36.08	$0.06	$0.33
	Yes	1.568	7.439	0.015	0.072	$7.14	$33.90	$0.07	$0.33

ANOVA tests were used to examine if the difference in mean values of each metric between demographic categories were different from 0. The sample used for the statistical test is denoted in parenthesis in the column description. A * denotes significance at the 5% level and ** denotes significance at the 1% level.

The number of ground meat units purchased was significantly lower among PBMA buyers (15.43) than the full sample (15.95), which resulted in significantly lower total expenditure on ground meat options (Table 2) among PBMA buyers compared to non-buyers. The average ground meat units and expenditure purchased by a buyer in the market was fairly constant across the two year span with a spike during the early stages of the COVID-19 pandemic in March of 2020 (Supplementary Fig. 3; Supplementary Fig. 4).

Households that are middle age (35–64), have more members (≥ 2), married, not college educated, employed, high income (> $100,000), white, not Hispanic, and have children present purchased and spent more on ground meat than households that are younger (< 35) or older (65 +), less members (< 2), single males or females, college educated, unemployed, low (< $45,000) or middle ($45,000–$99,999) income, minority race, Hispanic, and without children present (Table 4). Among the sample limited to PBMA buyers, households that are middle age (35–64), have more members, married, not college educated, employed, high income (> $100,000), non-Hispanic, and with children present purchased more ground meat units and had higher expenditure on average than households that are younger (< 35) or older (65 +), have less members, single males or females, college educated, unemployed, low (< $45,000) or middle ($45,000–$99,999) income, Hispanic, and without children present. One primary difference is that African-American households purchased the most units, but white households had the highest expenditure. Similar patterns are observed for the weekly household average expenditure and purchases of ground meat where households that are middle age (35–64), larger, married, not college educated, employed, higher income (> $100,000), white, non-Hispanic, and with children present had the highest weekly expenditures and units purchased.

**Table 4:** Ground meat units purchased and expenditure by demographic categories.

Variable	Category	Average number of aggregate meat units purchased (Full sample)	Average number of aggregate meat units purchased (Meat alternative buyers)	Average number of meat units purchased weekly (Full sample)	Average number of meat units purchased weekly (Meat alternative buyers)	Average total meat expenditure (Full sample)	Average total meat expenditure (Meat alternative buyers)	Average weekly expenditure on meat (Full sample)	Average weekly expenditure on meat (Meat alternative buyers)
Age	< 35	15.187**	14.155**	0.146**	0.136**	$101.63**	$84.46**	$0.98**	$0.81**
	35–64	16.551**	16.166**	0.159**	0.155**	$116.93**	$102.35**	$1.12**	$0.98**
	65 +	14.958**	13.986**	0.144**	0.134**	$106.86**	$90.16**	$1.03**	$0.87**
Household Size	1	10.257**	8.731**	0.099**	0.084**	$62.56**	$48.50**	$0.60**	$0.47**
	2	16.379**	15.542**	0.157**	0.149**	$117.17**	$99.52**	$1.13**	$0.96**
	3	19.001**	18.320**	0.183**	0.176**	$136.21**	$117.73**	$1.31**	$1.13**
	4	19.492**	21.275**	0.187**	0.205**	$141.17**	$135.02**	$1.36**	$1.30**
	5 +	20.121**	22.180**	0.193**	0.213**	$156.25**	$154.02**	$1.50**	$1.48**
Marital Status	Married	17.939**	17.949**	0.172**	0.173**	$131.07**	$117.34**	$1.26**	$1.13**
	Single Female	12.194**	11.414**	0.117**	0.110**	$77.47**	$65.21**	$0.74**	$0.63**
	Single Male	10.996**	8.102**	0.106**	0.078**	$69.82**	$46.48**	$0.67**	$0.45**
College Education	No	16.327**	16.096**	0.157**	0.155**	$117.34**	$103.87**	$1.13**	$1.00**
	Yes	15.422**	14.786**	0.148**	0.142**	$106.32**	$91.81**	$1.02**	$0.88**
Employed	No	14.872**	13.950**	0.143**	0.134**	$105.29**	$88.53**	$1.01**	$0.85**
	Yes	16.379**	15.890**	0.157**	0.153**	$115.71**	$100.62**	$1.11**	$0.97**
Income	< $45,000	14.416**	13.220**	0.139**	0.127**	$97.25**	$78.65**	$0.94**	$0.76**
	$45,000-$99,999	16.479**	15.626**	0.158**	0.150**	$117.46**	$98.70**	$1.13**	$0.95**
	$100,000 +	16.997**	17.208**	0.163**	0.165**	$124.61**	$114.29**	$1.20**	$1.10**
Race	White	16.238**	15.658**	0.156**	0.151**	$117.16**	$101.53**	$1.13**	$0.98**
	African American	15.994**	16.876**	0.154**	0.162**	$97.20**	$93.46**	$0.93**	$0.90**
	Other Minority	13.441**	12.151**	0.129**	0.117**	$94.27**	$77.50**	$0.91**	$0.75**
Hispanic	No	16.086**	15.632**	0.155**	0.150**	$113.50**	$98.66	$1.09**	$0.95
	Yes	14.462**	13.412**	0.139**	0.129**	$104.53**	$88.85	$1.01**	$0.85
Presence of children	No	15.316**	14.291**	0.147**	0.137**	$107.55**	$90.44**	$1.03**	$0.87**
	Yes	18.486**	19.696**	0.178**	0.189**	$133.53**	$125.14**	$1.28**	$1.20**

ANOVA tests were used to examine if the difference in mean values of each metric between demographic categories were different from 0. The sample used for the statistical test is denoted in parenthesis in the column description. A * denotes significance at the 5% level and ** denotes significance at the 1% level.

### Potential implications of COVID-19

Additionally, we analyzed if the potential effects of the COVID-19 pandemic on the units purchased and expenditures on ground meats and PBMAs among the full sample of households and PBMA buyers (Supplementary Table 3). We observed that among both the full sample and PBMA buyers that purchases of ground meats and PBMAs significantly increased after the beginning of the COVID-19 pandemic.

## Discussion and conclusions

This study sought to provide a deeper understanding of the market for PBMAs. From November 2018 to November 2020, expenditure shares for PBMAs, out of the ground meat market, increased from 4.22 to 6.29%. Expenditure shares declined sharply in the early stages of the COVID-19 pandemic in March and April of 2020 but quickly recovered. Our expenditure share estimates are larger than previous analysis, likely due to the fact that we limited our analysis to ground products^28,30,33,39^.

Another objective of our study was to identify consumer segments of PBMA purchasers by behavior. We identified that a sizable portion of ground meat consuming households purchased PBMAs, and that among PBMA purchasers, repeat-purchasing behavior was common (60.15%) but not universal. Novelty seeking was also prevalent (39.85%), indicating a sizable portion of households were potentially not satisfied with the developments in PBMAs. A vast majority of PBMA purchasing households bought ground meat options in addition to selecting some PBMAs. However, it is important to note that on average, PBMA purchasing households purchased fewer units and spent less on ground meat than households that only purchased ground meat options. Our evidence suggests that entering the PBMA market does not deter ground meat purchasing households from purchasing ground meat, as weekly units purchased and expenditure on ground meat products increased on average after the purchase of the first PBMA. These results show that PBMAs largely to do not deter meat demand amongst purchasing households and that rising PBMA market shares are not necessarily indicative of an expanding market since most purchasers of the products also purchase meats.

Similar previous studies from stated preference surveys, we identify that consumers of PBMAs are more likely to be younger (< 35), higher income (> $100,000), female, college educated, and of non-white minority racially^16,24–26,34,35^. Our additional contributions include the finding that repeat-purchasing of PBMAs was prevalent, especially among households with a younger (< 35), employed, and college educated head. Despite the prevalence of repeat-buying among PBMA buyers, a majority of households that purchased PBMAs bought both ground meat and PBMAs, which is consistent with Zhao et al. (2022) indicating that PBMAs may be complementary to beef. This phenomenon of continuing ground meat purchases provides some evidence that PMBAs are not leading to substitution away from ground meat options^17–20^. The high prevalence of novelty seeking behavior also shows that PBMAs are not leading to substitution away from meats after purchasing PBMAs. This finding also suggests additional evidence that even though interest and market share have increased surrounding PBMAs, that meat markets and their supply chain are not significantly affected by the PBMA market^40^.

Our additional insight into consumer segments is observing differences in household expenditures on PBMAs and ground meats. We found that younger (< 35), single male, college educated, employed, high income (> $100,000), and African-American households on average had the highest weekly expenditures of PBMAs. When considering only buyers of PBMAs we observe few significant differences between households in expenditure other than in marital status, which potentially indicates that households that are purchasers of PBMA exhibit similar preferences across demographic groups. This is not true of ground meat purchasers, where many significant differences occurred in patterns of expenditure. Households with more members, higher income, married, and with children present income had the highest expenditure.

Finally, we also identified that the COVID-19 pandemic had a significant effect on the units purchased and household expenditure of ground meats and PBMAs. We observed that households on average, purchased more units of both ground meats and PBMAs after the beginning of the COVID-19 pandemic. These results are consistent with general estimates on food and grocery spending, which indicate that expenditures and volumes of food purchased at home increased drastically at the onset of the COVID-19 pandemic^41^.

PMBA sales grew from November 2018 to November 2020. However, some recent reports have suggested declining sales^42^. Our results foreshadow this decline in sales with the sizable portion of households engaging in novelty seeking behavior as well as the general trend of declining market entry amongst households. A particularly interesting finding is that few significant differences were measured in mean purchases and expenditures of PBMAs between purchasing households, which indicates that demographic differences are not evident among buyers of PBMAs indicating they may have similar preference structures. Despite the increase in market share and repeat-purchases among PBMA purchasing households, entering the PBMA market did not decrease household purchases of ground meat. Future research can include insight into important product characteristics consumers consider when purchasing PBMAs. Another interesting segment of this market is food away from home. Despite reports of declining sales multiple restaurant chains, such as, Kentucky Fried Chicken (KFC) have recently introduced meatless chicken nuggets^43^, which indicate that food service providers still see PBMAs as viable products. This market is still in its infancy and will likely substantially evolve in time.

Many questions still remain with respect to PBMAs. One primary issue are the questions surrounding the healthiness of the new novel PBMAs for long term human health, despite having similar macronutrient values to the beef options they are simulating^44–46^. It is also unclear as to the effects of the novel PBMAs on the environment. The companies producing the products have released favorable life cycle analyses regarding these products^17,18^. Despite the favorable life cycle analysis results, some have estimated suggest that changes in the prices of PBMAs do little to affect the beef supply chain, thus potentially only inducing minor environmental and climate benefits^40^.

## Methods

### Data

This study utilizes the Information Resources, Inc. (IRI) household scanner panel dataset. Households who made at least one meat or PBMA purchase from November 2018 to November 2020 were included. The resulting data set consists of individual-transaction data from 38,966 households. Each entry in the dataset is recorded at a universal product code (UPC) level. The dataset includes UPCs of the primary categories of beef, poultry, seafood, pork, PBMAs, turkey, etc. Each UPC code is attached to a product description which includes characteristics such as the brand, processing method, packaging, and size. Additionally, each UPC represents a specific set of product characteristics which include the brand, type of meat, fat content, weight, etc. We aggregated purchases to the weekly level, implying 104 weeks of observations for each of the 38,966 households.

The PBMAs did not include non-patty or non-ground substitutes such as sausage link substitutes. The PBMAs studied resemble ground meat products and are theoretically the closest substitute. Thus, we restricted analysis to purchases of ground meat options (ground beef, ground chicken, ground turkey, or ground/crumble/patty), and PBMA patties and burgers. The ground meat products include categories of ground beef, beef burgers, ground turkey, turkey burgers, ground chicken, soy burgers, and newer forms of PBMAs. The total number of transactions in the dataset are 505,262 consisting of 439 UPCs. The UPCs that remained in the dataset were those were purchased on 50 or more occasions in the span of the dataset. Many UPCs that were purchased on 50 or less occasions were unclear as to what the products specifically were, and thus were not considered for this analysis.

In addition to product characteristics, the demographics of each household are known. The demographics considered are the age of the household head (young (< 35), middle age (35–64), old (65 +)), the number of individuals in the household, marital status (married, single female, single male), education level (college degree or not), employment status (employed or not), income level (low (< $45,000), middle ($45,000-$99,999), high (> $100,000)), race (white, African-American, other races), Hispanic, and the presence of children. Due to the nature of the data, some household entries contain characteristics of both a female and male head of household. In these cases, the female characteristics are considered as the primary characteristics when a female household head is present due to the fact that a majority of primary shoppers in household are females^47^. Female characteristics were used when applicable for the age of the household head, and the education level as the other demographic characteristics were used to describe the household in general without taking into account the specific heads of household. Supplementary table 1 details the household demographic breakdown.

After identifying the relevant demographics and UPCs we aggregate the products using indicator variables for ground beef, turkey, chicken, and PBMAs. We aggregate the purchases for each of these product types for each individual household for each week over the span of two years. These aggregations formulate a dataset of 38,966 households that purchased at least one of the UPCs in the product categories of ground beef, turkey, chicken, and PBMAs. Next, we identified the households who purchased at least one PBMA over the span of the dataset, which added up to 7761 households.

### Data analysis

Our first market analysis is the expenditure share of PBMAs relative to other ground meats over the two year span of the dataset. We estimate the expenditure share by taking the sum of dollars spent on a particular ground meat option in a given week and divided that by the total expenditure on ground meat and PBMAs in a given week. Additionally, we estimate the market expenditures and units purchased for ground meats and PBMAs by taking the total expenditure and units purchased in a given week and divide that by the number of households who purchased a product in that specific week.

Next we estimate an overview of purchasing behavior by both the full sample and PBMA consumers estimating the share of PBMA buyers, the share of households that purchased PBMAs on more than one occasion, the share of households that did not purchase ground meat, the share of households who only purchased ground meat and no PBMAs, and the share of households who purchased both ground meat and PBMAs. We estimate the share of households that purchased PBMAs by denoting a household as a buyer if they purchased at least one PBMA over the span of two years. This provides the smaller subset of the sample of PBMA buyers. The share of households who made repeat purchases is estimated by examining if a household if made purchases of PBMAs on multiple occasions in the dataset. If they did make multiple purchases they were assigned an indicator variable for engaging in habit forming behavior. Next, we estimated the share of households that did not purchase ground meat. These estimates were calculated by assigning a dummy variable to a household that purchased at least 1 one ground meat item over the span of the dataset. If they received a “0” for this indicator they were included in the share that did purchase ground meat. Using the indicator variable for ground meat buyers and PBMA buyers, we also estimate the share of households who purchased ground meat only, and the share of households that purchased both ground meat and PBMAs.

After estimating the overall shares of household behaviors, we estimate the average aggregate number of units purchased, average number of weekly units purchased, average total expenditure, and average weekly expenditure for both PBMAs and ground meat. We estimate the number of units purchased by calculating the sum of units of ground meat and PBMAs purchased by a household. The weekly units are estimated by dividing the total number of units purchased by the 104 weeks of data collection. The same process is repeated for the ground meat and PBMA expenditures.

The final metric is to estimate if ground meat units and expenditures changed after the household entered the PBMA market by making their first purchase. We estimate the average weekly consumption of ground meat products by first finding the week that a household made their first purchase of a PBMA. Next we took the sum of ground meat units and expenditure on ground meat products before and after the first purchase occurred. After taking the sum of units and expenditures, we divide the sums by the number of weeks before and after the first purchase. The averages are then compared in a paired t-test to examine if purchasing patterns changed amongst households after the first PBMA purchase.

After the estimating the general behavioral overview of the households in the market, we analyzed the consumer segments and purchasing patterns by demographic groups. First we estimated the consumer segments, which were the share of PBMA buyers, the habit forming buyers who purchased on more than one occasion, the share of households who did not purchase PBMAs, the share of households who purchased both ground meat and PBMAs, and the share of households that did not purchase ground meat. To test the differences a chi-squared test was used where the null hypothesis is that no association is detectable between the binary outcome variable and the demographics.

Next, we estimated the demographic differences for the aggregate and weekly number of units purchased and expenditures for both ground meat options and PBMAs. Due to these variables being continuous outcomes, we employed an analysis of variance (ANOVA) test with a null hypothesis that the difference in means between demographic groups for these metrics were equal to 0.

The final metric we estimated was if household purchases and expenditures of ground meats and PBMAs after the beginning of the COVID-19 pandemic. We estimated the average number of units purchased and expenditures prior to March 1, 2020 and after. This date was chosen as it resembled the month in which the early stages of the economic disruptions from COVID-19 began in the food sector^41^. We tested the differences in the averages with a paired t-test before and after the beginning of the pandemic.

We confirm that all methods were carried out in accordance with the relevant guidelines from Information Resources Inc. (IRI) and Purdue University. Due to the nature of the data the approval to conduct research with this data was given by a signed contract with Information Resources Inc. (IRI). All respondents give consent to Information Resources Inc.

## Supplementary Information

Supplementary Information.

## Data Availability

Due to contractual obligations with Information Resources, Inc. (IRI), we are not at liberty to share the data.
