# Supplementary material for: Most plant-based meat alternative buyers also buy meat: an analysis of household demographics, habit formation, and buying behavior among meat alternative buyers
Source: Sci Rep. 2022 Jul 29;12:13062. doi: 10.1038/s41598-022-16996-5 (PMC9336146; doi:10.1038/s41598-022-16996-5)
Supplement: Supplementary file 1 — Supplementary Information. [file 41598_2022_16996_MOESM1_ESM.pdf]

# Most plant-based meat alternative buyers also buy meat: An analysis of household demographics, habit formation, and buying behavior among meat alternative buyers.

**Zachary T. Neuhofer[1*], and Jayson L. Lusk[1]**

May 11, 2022

**Keywords:** plant-based, meat alternatives, demographics, purchasing patterns, repeat purchases

[1]Department of Agricultural Economics, Purdue University, West Lafayette, IN, USA

[*]Corresponding Author. Email: zneuhofe@purdue.edu

Supplementary Figure 1: Average Market Expenditure on Plant-Based Meat Alternatives by week

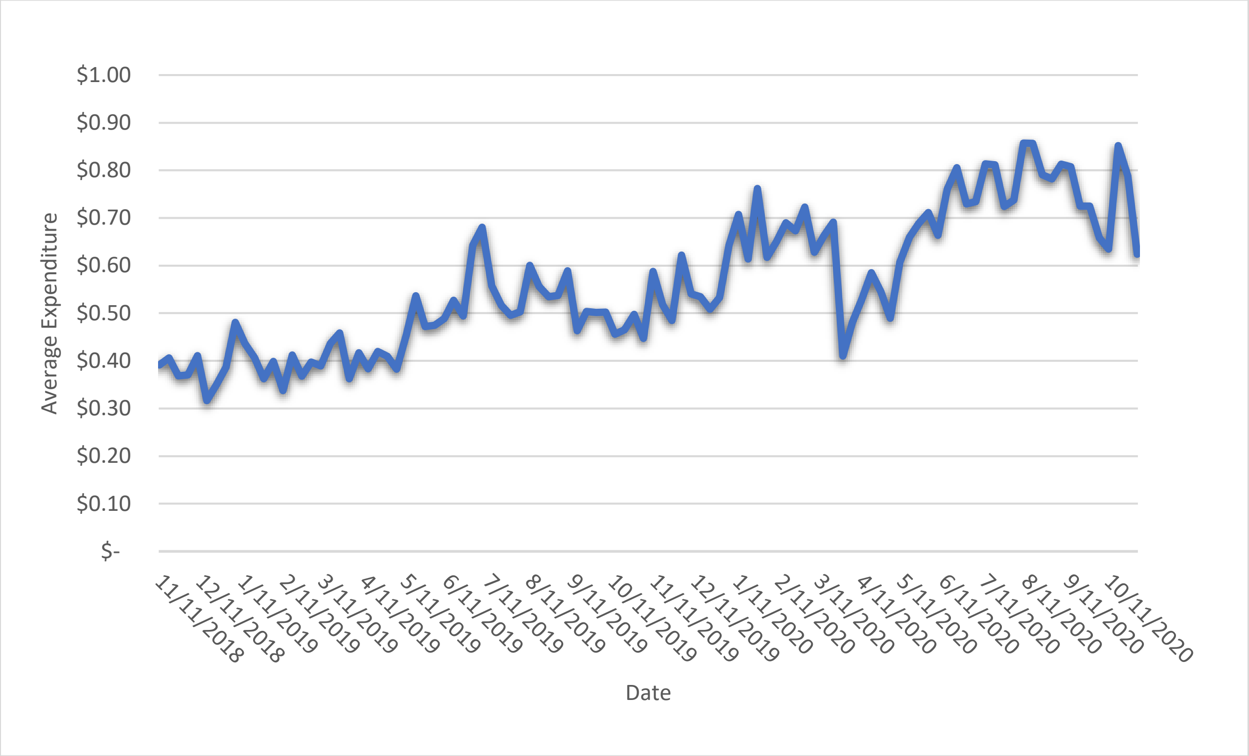

Supplementary Figure 2: Average Market Plant Based Meat Alternative Units Purchased by Week

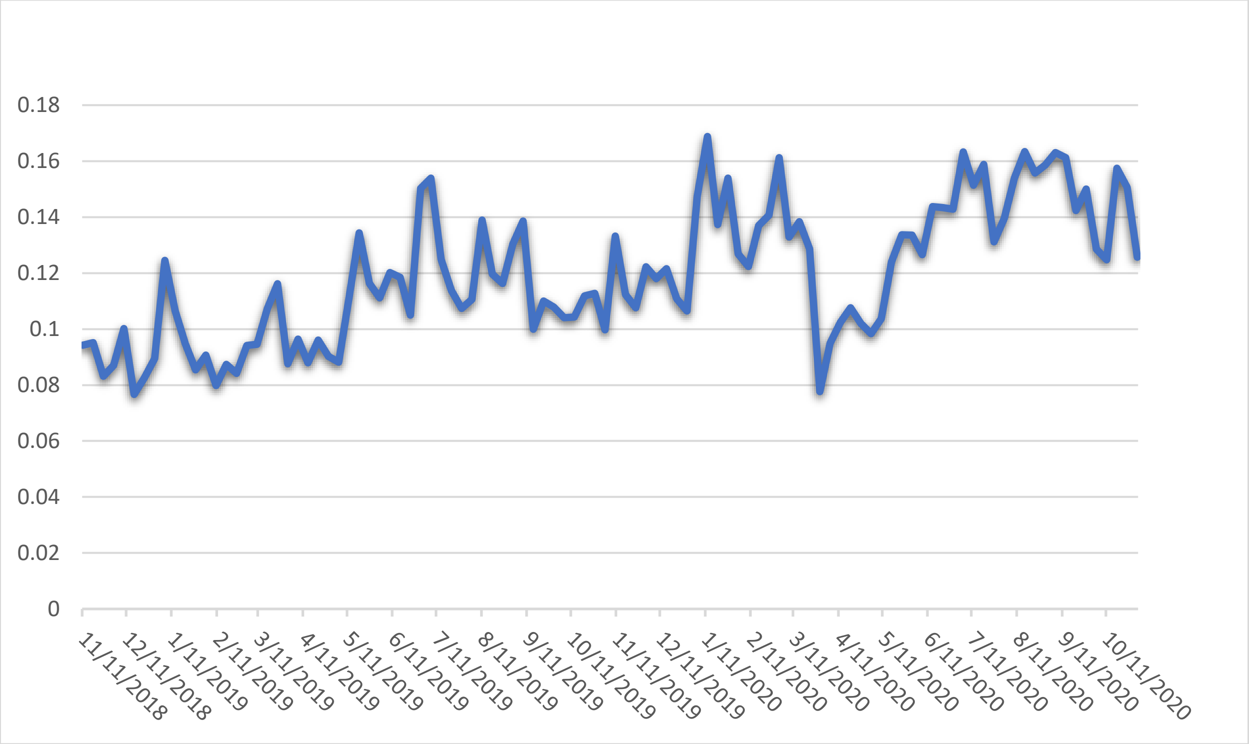

Supplementary Figure 3: Average Market Expenditures on Ground Meat

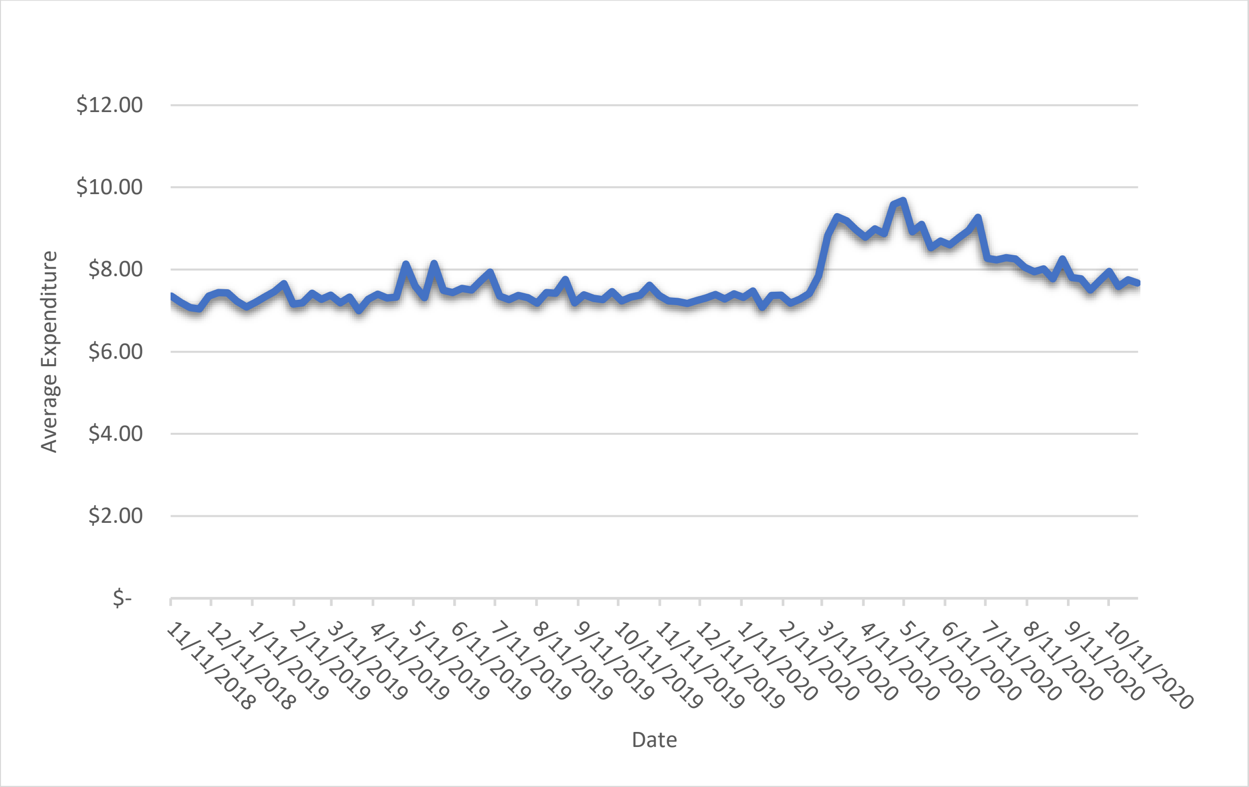

Supplementary Figure 4: Average Market Ground Meat Units Purchased by Week

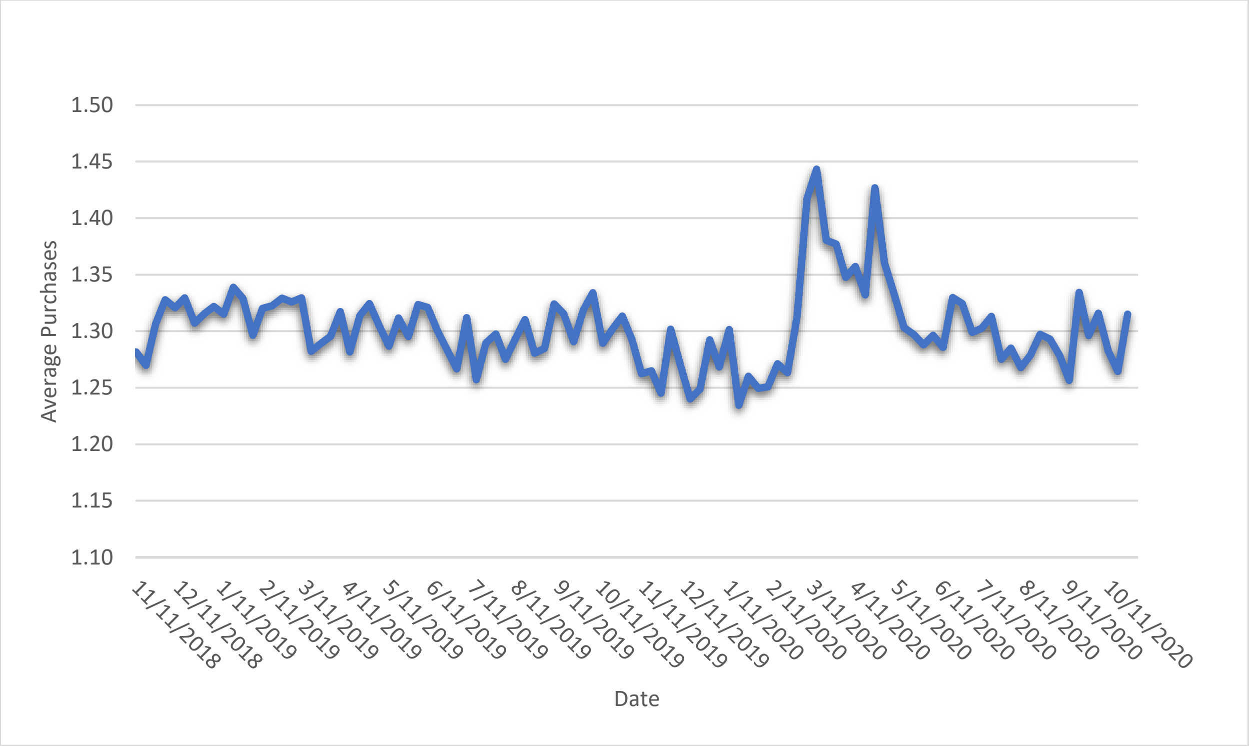

Supplementary Figure 5: Number of Households First Purchasing a Plant-Based Meat Alternative

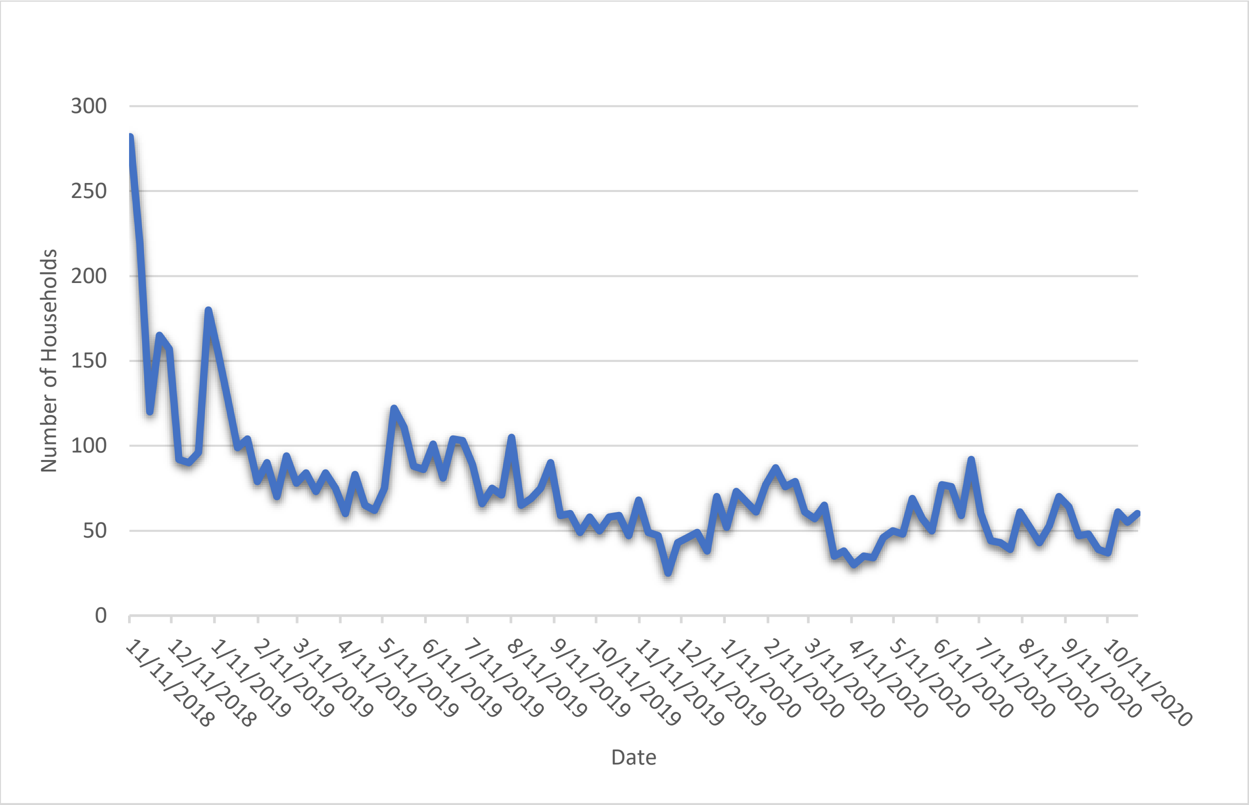

Supplementary Table 1: Sample Demographics

| Variable | Category | Full Sample | Meat Alternative Buyers | Meat Only Buyers |
|---|---|---|---|---|
| Age | <35 | 5.73% | 7.23% | 5.35% |
| | 35-64 | 61.43% | 65.85% | 60.33% |
| | 65+ | 32.84% | 26.92% | 34.31% |
| Household Size | 1 | 22.78% | 24.17% | 22.44% |
| | 2 | 45.49% | 42.56% | 46.22% |
| | 3 | 13.73% | 14.60% | 13.52% |
| | 4 | 10.83% | 11.79% | 10.59% |
| | 5+ | 7.16% | 6.88% | 7.23% |
| Marital Status | Married | 66.76% | 64.82% | 67.24% |
| | Single Female | 26.57% | 28.63% | 26.06% |
| | Single Male | 6.66% | 6.88% | 6.69% |
| College Education | No | 58.31% | 49.45% | 60.51% |
| | Yes | 41.69% | 50.55% | 39.49% |
| Employed | No | 28.48% | 23.50% | 29.72% |
| | Yes | 71.52% | 76.50% | 70.28% |
| Income | <$45,000 | 31.43% | 26.50% | 32.66% |
| | $45,000-$99,999 | 45.67% | 45.34% | 45.75% |
| | $100,000+ | 22.90% | 28.15% | 21.59% |
| Race | White | 79.26% | 75.45% | 80.21% |
| | African American | 11.43% | 13.46% | 10.92% |
| | Other Minority | 9.31% | 11.08% | 8.87% |
| Hispanic | No | 91.58% | 91.06% | 91.71% |
| | Yes | 8.42% | 8.94% | 8.29% |
| Presence of children | No | 80.02% | 78.86% | 80.31% |
| | Yes | 19.98% | 21.14% | 19.69% |
| Total | | 38,966 | 7761 | 31,205 |

Supplementary Table 2: Market Overview Metrics

| Metric | Sample | Sample Size | Share or Average |
|---|---|---|---|
| | *Behavioral overview* | | |
| Share of meat alternative buyers | Full Sample | 38,966 | 19.92% |
| Share of households who did not purchase meat alternatives | Full sample | 38,966 | 80.08% |
| Share of meat alternative buyers who purchased them on more than one occasion | Full Sample | 38,966 | 12.03% |
| | Meat Alternative Buyers | 7,761 | 60.15% |
| Share of households that did not purchase meat | Full Sample | 38,966 | 2.79% |
| | Meat Alternative Buyers | 7,761 | 14.51% |
| Share of meat alternative buyers who purchased meat and meat alternatives | Full Sample | 38,966 | 17.12% |
| | Meat Alternative buyers | 7,761 | 85.97% |

Supplementary Table 3: Ground Meat and Meat Alternative Purchases and Expenditure Before and After the Beginning of the COVID-19 Pandemic

| Metric | Sample | Sample Size | Average |
|---|---|---|---|
| Average units of meat purchased weekly prior to the beginning of the COVID-19 pandemic | Full Sample | 38,966 | 0.14++ |
| Average units of meat purchased weekly after the beginning of the COVID-19 pandemic | Full Sample | 38,966 | 0.16++ |
| Average units of PBMAs purchased weekly prior to the beginning of the COVID-19 pandemic | Full Sample | 38,966 | 0.01++ |
| Average units of  purchased weekly prior to the beginning of the COVID-19 pandemic | Full Sample | 38,966 | 0.02++ |
| Average units of ground meat purchased weekly prior to the beginning of the COVID-19 pandemic | Meat Alternative Buyer | 7,761 | 0.14++ |
| Average units of ground meat purchased after the beginning of the COVID-19 pandemic | Meat Alternative Buyer | 7,761 | 0.15++ |
| Average units of meat alternatives purchased weekly prior to the beginning of the COVID-19 pandemic | Meat Alternative Buyer | 7,761 | 0.06++ |
| Average units of meat alternatives purchased after the beginning of the COVID-19 pandemic | Meat Alternative Buyer | 7,761 | 0.08++ |
| Average weekly expenditure on ground meats prior to the beginning of the COVID-19 pandemic | Full Sample | 38,966 | $0.99++ |
| Average weekly expenditure on ground meats after  the beginning of the COVID-19 pandemic | Full Sample | 38,966 | $1.25++ |
| Average weekly expenditure on meat alternatives prior to the beginning of the COVID-19 pandemic | Full Sample | 38,966 | $0.05++ |
| Average weekly expenditure on meat alternatives after  the beginning of the COVID-19 pandemic | Full Sample | 38,966 | $0.08++ |
| Average weekly expenditure on ground meats prior to the beginning of the COVID-19 pandemic | Meat Alternative Buyers | 7,761 | $0.87++ |
| Average weekly expenditure on ground meats after  the beginning of the COVID-19 pandemic | Meat Alternative Buyers | 7,761 | $1.08++ |
| Average weekly expenditure on meat alternatives prior to the beginning of the COVID-19 pandemic | Meat Alternative Buyers | 7,761 | $0.28++ |
| Average weekly expenditure on meat alternatives after  the beginning of the COVID-19 pandemic | Meat Alternative Buyers | 7,761 | $0.42++ |

A + denotes a paired T-test to compare the difference in means of two different variables with the null hypothesis $\mu_{after} - \mu_{before}$. A + denotes significance at the 5% level and a ++ denotes significance at the 1% level.
